# NEUROTICISM PREDICTS INFORMANT-REPORTED COGNITIVE DECLINE THROUGH HEALTH BEHAVIORS

**DOI:** 10.1093/geroni/igz038.3115

**Published:** 2019-11-08

**Authors:** Rachel D Best, Patrick J Cruitt, Patrick L Hill

**Affiliations:** Washington University in St. Louis, St. Louis, Missouri, United States

## Abstract

Our current study attempts to better understand the relationship between personality and cognitive decline. In this study, we analyzed whether health behaviors act as mediating variables for the relationship between personality and cognitive decline. Additionally, we were interested to see how personality influences different health behaviors, and which health behaviors in particular are predictive of cognitive decline. In addition to analyzing the composite score of health behaviors in relation to personality and cognitive decline, we analyzed each of its four components (wellness maintenance, accident control, traffic risk, and substance abuse; Vickers, Conway & Hervig, 1990). To measure cognitive decline, we used the Ascertain Dementia Eight Item Scale, an informant-report screening measure. Personality has consistently been linked to cognitive decline (Curtis, Windsor & Soubelet, 2015; Chapman, Duberstein, Tindle, Sink, Robbins, Tancredi & Franks, 2013; Low, Harrison & Lackersteen, 2013), but has not yet been analyzed with an informant report measure. Informant-report may be more reliable than self-report when measuring cognitive decline because participants who exhibit cognitive impairment may not be equipped or willing to report about their own cognitive ability. We found that neuroticism significantly predicted informant-reported cognitive decline and that this relationship was mediated by health behaviors, specifically, wellness maintenance. Wellness maintenance was the only category of health behavior that predicted informant-reported cognitive decline. Surprisingly, conscientiousness was unrelated to informant-reported cognitive decline as were extraversion, agreeableness, and openness to experience.

